# Origins and diversity of macrophages in health and disease

**DOI:** 10.1002/cti2.1222

**Published:** 2020-12-20

**Authors:** Gopalkrishna Sreejit, Andrew J Fleetwood, Andrew J Murphy, Prabhakara R Nagareddy

**Affiliations:** ^1^ Division of Cardiac Surgery Department of Surgery The Ohio State University Wexner Medical Center Columbus OH USA; ^2^ Division of Immunometabolism Baker Heart and Diabetes Institute Melbourne VIC Australia

**Keywords:** innate immunity, macrophage diversity, macrophage functions, macrophage niche, macrophage origins

## Abstract

Macrophages are the first immune cells in the developing embryo and have a central role in organ development, homeostasis, immunity and repair. Over the last century, our understanding of these cells has evolved from being thought of as simple phagocytic cells to master regulators involved in governing a myriad of cellular processes. A better appreciation of macrophage biology has been matched with a clearer understanding of their diverse origins and the flexibility of their metabolic and transcriptional machinery. The understanding of the classical mononuclear phagocyte system in its original form has now been expanded to include the embryonic origin of tissue‐resident macrophages. A better knowledge of the intrinsic similarities and differences between macrophages of embryonic or monocyte origin has highlighted the importance of ontogeny in macrophage dysfunction in disease. In this review, we provide an update on origin and classification of tissue macrophages, the mechanisms of macrophage specialisation and their role in health and disease. The importance of the macrophage niche in providing trophic factors and a specialised environment for macrophage differentiation and specialisation is also discussed.

## Graphical abstract

In this review, we provide a clear, comprehensive update on monocyte/ macrophage ontogeny, differentiation and function.

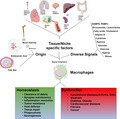

## Introduction

Macrophages were first identified by Metchnikoff in 1882 when he observed phagocytes surrounding and trying to devour a rose thorn introduced into the transparent body of a starfish larva.^1^, ^2^ Metchnikoff also identified major roles for these phagocytes in host resistance against infections, phagocytosis of unwanted cells during development, injury and repair. Macrophages have subsequently been shown to initiate and shape the adaptive immune system and in general acting as an inflammation rheostat. Macrophages achieve this by processing and presenting antigens to T cells^3^ and by integrating multiple signals from a repertoire of cell surface and cytoplasmic pattern recognition receptors.^4^

Macrophages are the first immune cells to appear in an organism’s development and are essential during the early stages of development.^5^ Tissue macrophages also play a crucial role in homeostasis,^6^, ^7^ wound healing^8^ and tissue regeneration.^9^, ^10^ The wide variety of macrophage functions partly arise because of their ability to sense and sample the local tissue environment and via expression of specific transcription factors and enhancer‐associated histone modifications unique to a local microenvironment.^11^, ^12^ Macrophages are also able to make extensive changes to their intracellular metabolism in response to environmental and inflammatory cues.^13^ Unfortunately, aberrant macrophage function is strongly associated in the pathogenesis of disease states such as fibrosis, obesity and cancer.^14^ In this review, we discuss our current understanding of the ontogeny of tissue‐resident macrophages, the interaction of macrophages with components of the tissue niche and how these interactions shape macrophage function. We also discuss the links between cellular metabolism and macrophage phenotype, the contribution of monocytes to the maintenance of tissue macrophage populations and how monocyte‐derived macrophages differ from embryo‐derived macrophages.

## Macrophage ontogeny and the mononuclear phagocyte system

Macrophages were classified as part of mononuclear phagocyte system (MPS) along with monocytes and dendritic cells (DCs) in the mid‐1970s.^15^ According to the description of the MPS, tissue macrophages were considered fully differentiated cells that were constantly being replenished by circulating monocytes.^16^ The concept of MPS is supported by *in vitro* studies showing monocyte differentiation into macrophages and *in vivo* adoptive transfer of monocytes under inflammatory conditions showing recruitment and conversion to macrophages in the peritoneal cavity.^17^ However, several studies in humans and mice have contradicted the non‐dividing, terminally differentiated, circulation‐dependent ontogeny of tissue macrophages. In congenic parabiotic mice, which share the same circulation and have a mixed population of lymphocytes and monocytes in the blood, the macrophage populations in the brain^18^, ^19^ and epidermis^20^ do not mix even after a year of parabiosis. Moreover, histological approaches have demonstrated the presence of macrophages before the establishment of definitive haematopoiesis that gives rise to monocytes.^21^, ^22^, ^23^ Several human studies have further supported the circulation‐independent origin of tissue macrophages. For example, patients with severe monocytopenia have normal numbers of macrophages in the epidermis (Langerhans cell, LC)^24^, ^25^ and host LCs remained in patients who received sex‐mismatched allogeneic bone marrow transplants.^26^, ^27^ Donor LCs can also be detected for years in recipients of human limb graft.^28^ Donor macrophages also self‐maintain for years in the transplanted heart,^29^ liver^30^ and lungs.^31^, ^32^, ^33^ Despite these findings, more work is needed to understand the origin of tissue macrophages in humans. Much of our current knowledge of tissue macrophage ontogeny comes from mouse models. It should be noted that whilst these models are extremely useful, they have inherent limitations around life span and environmental exposure that may not reflect the situation in humans.

The embryonic origin of tissue macrophages has also been confirmed by Cre‐LoxP approaches. The chemokine receptor, CX3CR1, is prominently expressed in the MPS.^34^ Using CX3CR1Cre:R26‐YFP reporter mice that display constitutive Cre activity in CX3CR1^+^ cells and drug‐induced activation of Cre in CX3CR1CreER:R26‐YFP mice, it has been established that most tissue macrophages are generated prenatally that self‐renew in peripheral tissues during adulthood at least in the absence of challenge.^17^, ^35^ These observations led to the conclusions that tissue‐resident macrophages are not solely derived from haematopoietic stem cells (HSCs) or BM‐derived progenitors but also derived from local or embryonic precursors.^36^ This has led investigators to more thoroughly explore the embryonic origin of macrophages.

## Embryonic macrophages

**Figure 1:**
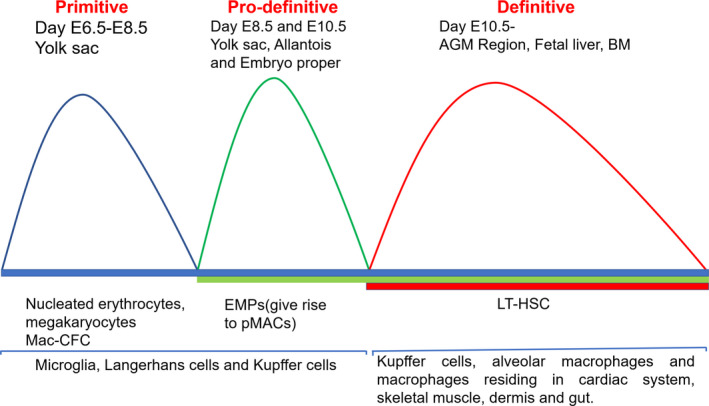
Origin of macrophages. Myeloid cells including macrophages arise from three successive haematopoietic waves, referred to as primitive, pro‐definitive and definitive. The primitive programme starts at embryonic days 6.5 (E6.5)‐E8.5 in the blood islands of the extraembryonic yolk sac (YS) and gives rise to nucleated erythrocytes, megakaryocytes and Mac‐CFCs. The pro‐definitive wave starts at E8.5 and E10.5 in the yolk sac, allantois and embryo proper and gives rise to erythroid and myeloid progenitors (EMPs). Primitive and pro‐definitive phases contribute to microglia, Langerhans and Kupffer cells. The third wave of definitive haematopoiesis starts at E10.5 from the aorta–gonad–mesonephros region (AGM) region and gives rise to LT‐HSC. They migrate to the foetal liver and definitive haematopoiesis shifts to BM around E17.5. Definitive haematopoietic stem cells give rise to Kupffer cells and alveolar macrophage tissue residing in cardiac system, skeletal muscle, dermis and gut.

Myeloid cells including macrophages arise from three successive haematopoietic waves, referred to as primitive, pro‐definitive and definitive phases, respectively^37^ (Figure 1). The primitive programme is independent of the transcription factor c‐Myb and starts at embryonic days 6.5 (E6.5)–E8.5 in the blood islands of the extraembryonic yolk sac (YS). This phase gives rise to bipotent progenitors for nucleated erythrocytes and megakaryocytes and a progenitor restricted to the macrophage lineage (Mac‐CFC).^38^, ^39^, ^40^, ^41^, ^42^ The c‐Myb‐independent, pro‐definitive wave occurs in different sites of the embryo (YS, allantois and embryo proper) and gives rise to erythroid and myeloid progenitors (EMPs) between E8.5 and E10.5.^43^, ^44^ Unlike long‐term haematopoietic stem cells (LT‐HSCs), EMPs do not have long‐term repopulating capacity and develop into macrophages through a CX3CR1 expressing intermediate population called p‐Macs.^45^ EMPs give rise to p‐Macs without passing through the monocyte stage as evidenced by the lack of peroxidase activity (a signature feature of monocytes) and the presence of a core macrophage transcriptional programme occurring in p‐Macs.^45^ EMPs and p‐Macs expand in the YS and then traffic towards foetal liver up until E14.5, where they serve as a reservoir for macrophages throughout embryogenesis.^43^, ^44^ EMP progeny seed different tissues in the embryo and may become life‐long tissue‐resident macrophages.^37^, ^46^, ^47^, ^48^ Brain microglia are a prototypical primitive macrophage generated in the YS, which are maintained throughout adult life by virtue of their longevity and limited self‐renewal capability without input from definitive haematopoiesis.^18^, ^19^, ^49^ EMP‐derived macrophages contribute to embryonic development and tissue remodelling through phagocytosis of unwanted and obsolete cell structures and cells.^50^, ^51^

Macrophages also act as cellular chaperones for tissue vascularisation.^52^ Mouse mutants lacking macrophages during embryonic development, because of deficiency of colony‐stimulating factor 1 receptor (CSF‐1R also known as CD115) or the transcription factor PU.1, display growth retardation and perinatal mortality.^53^, ^54^ The transcription factor c‐Myb is not needed for primitive haematopoiesis but is required for definitive haematopoiesis.^55^ This was shown in Myb mutant mouse embryos, where impairment in definitive haematopoiesis was seen,^56^, ^57^ but tissue‐resident macrophages in the brain (microglia), skin (LCs) and liver (Kupffer cells; KC) were unaffected.^58^ Similarly, Myb mutant zebrafish develop tissue macrophage populations in the absence of definitive haematopoiesis.^55^ The third wave of c‐Myb‐dependent definitive haematopoiesis starts at E10.5 from the aorta–gonad–mesonephros region (AGM) region and gives rise to LT‐HSC. They migrate to the foetal liver and definitive haematopoiesis shifts to the bone marrow (BM) at around E17.5.^38^, ^59^, ^60^ Definitive HSCs arising from the AGM region at around E10.5^61^ give rise to KCs and alveolar macrophages. HSC activity then peaks in the foetal liver at around E16.5 and gives rise to tissue macrophages residing in the cardiac system, skeletal muscle, dermis and the gut before shifting to BM.^62^ The BM then remains the major site of haematopoiesis in adult life.

## Primitive vs. definitive origin of macrophages

Despite the general agreement that most tissue macrophages have an embryonic origin, the exact contributions of primitive and definitive haematopoiesis to embryo‐derived adult tissue macrophage populations remain unclear. All tissue macrophages may arise from Myb and HSC‐independent lineage without going through a monocyte intermediate.^63^ These macrophages can seed various locations and give rise to bonafide long‐lived tissue macrophages. For example, the BM contains precursor cells that give rise to LC and microglia.^64^, ^65^ Macrophages may also arise from definitive haematopoiesis in foetal liver through a monocyte intermediate.^19^ Pulse labelling of myeloid precursors in the Runx1CreER mouse has enabled researchers to determine the relative contributions of YS and foetal liver to tissue macrophage populations. Runt‐related transcription factor 1 (Runx1) expression is restricted to the extraembryonic YS between E6.5 and E8.^19^, ^66^ Inducible CreER reporter gene expression driven by Runx1 has established considerable input from foetal liver‐resident precursors to lung, dermis and spleen macrophages^19^ with the exception of microglia that originate solely from the yolk sac. Most tissue macrophages except microglia lose their Runx1^+^ labelling in adult tissues suggesting that they are replaced by non‐labelled precursors before birth.^19^, ^67^, ^68^ Contributions of YS versus foetal liver‐derived precursors, however, vary between tissue macrophage compartments. For example, heart‐resident, cardiac macrophages are derived from both YS‐derived and foetal liver‐derived progenitors,^69^ while adult LC^68^ and adult lung alveolar macrophages^70^ mainly originate from foetal liver‐derived monocytes. Despite the results from the Runx1^+^ mice, the origin of cells arising from foetal liver is less well‐defined because foetal liver is itself seeded by YS precursors.^38^, ^39^

The revised concept of MPS now accommodates two independent origins of tissue macrophages. Embryonic macrophages are established prenatally and self‐maintain independent of any haematopoietic input,^17^, ^19^, ^58^, ^69^, ^70^, ^71^, ^72^ whereas adult‐derived macrophages develop from tissue‐infiltrating monocytes, have a limited lifespan and are associated with pathological inflammatory reactions. Both types of macrophages seem to co‐exist in tissues, whether they have different behaviour based on ontogeny or are made functionally homogenous by the tissue environment remains to be seen.

## Macrophage subsets

Macrophages are a highly heterogenous population of cells. The initial classification of macrophages into M1 and M2 subsets was based on macrophages isolated from C57BL/6 mice and Balb/c mice. Macrophages from C57BL/6 mice have a Th1‐dominated immune response and, when challenged with LPS and IFN‐γ, produce nitric oxide (NO) from arginine via iNOS.^73^ Macrophages from Balb/c mice have a Th2‐dominated immune response and, when challenged by LPS and IFN‐γ, produce ornithine via arginase.^73^ C57BL/6 mice carry a deletion in the promoter of *Slc7a2*, the key arginine transporter in macrophages causing large differences in arginine utilisation between C57BL/6 and BALB/c mice.^74^ Categorisation of macrophages into M1 and M2 subsets based on arginine metabolism fits neatly with the inflammation vs. resolution functions of macrophages. Macrophages producing NO inhibit/kill pathogens or nearby cells, while ornithine promotes cell proliferation and wound healing. M1/M2 classification has also been used to define macrophage polarisation states. LPS and IFN‐γ induce M1 macrophages in a STAT‐1 and aerobic glycolysis‐dependent manner,^75^ while IL‐4 induces M2 macrophages in a STAT6 and fatty acid oxidation (FAO)‐dependent manner.^76^, ^77^ Currently, M1/M2 macrophages are divided based on the expression of specific markers; M1 macrophages express CD68, TNF‐α, iNOS, IL‐1β and IL‐12, while M2 macrophages express arginase 1, transforming growth factor (TGF)‐β, CD163 (cluster of differentiation 163), mannose receptor 1, CD206, Rtnla, IL‐10, VEGF and Ym1.^78^, ^79^ M1 macrophages produce pro‐inflammatory cytokines (TNF‐α, IL‐12, IL‐27 and IL‐23), chemokines (CXCL11, CXCL9 and CXCL10) and matrix‐metalloproteinases (MMP‐1, 2, 7, 9, 12) and demonstrate enhanced antigen presentation and generation of reactive oxygen species. In contrast, macrophages stimulated with IL‐4 and IL‐13 show an anti‐inflammatory and reparative profile.^80^ M2 macrophages produce anti‐inflammatory cytokines (IL‐10), chemokines (CCL17)^81^, ^82^ and growth factors (VEGF, TGF‐β). Together, these mediators promote tissue remodelling and repair by stimulating extracellular matrix production by fibroblasts, cell proliferation and angiogenesis.

The classification of macrophages into M1/M2 groups based on well‐defined stimuli does not model the infinitely more complex tissue milieu where macrophages (potentially of different origin) would be exposed to multiple signals in different sequential order. Nevertheless, macrophages have been classified into subgroups within the M1‐M2 range as M2a, M2b, M2c and Mox macrophages.^79^, ^83^, ^84^, ^85^ Given the phenotypic diversity of macrophage populations *in vivo*, the relevance of the M1‐M2 paradigm may be minimal. For example, one study acquired a data set of 299 macrophage transcriptomes in response to diverse activation signals.^85^ In another study, CyTOF analysis of renal cancer macrophages identified 17 different subsets.^86^ A plethora of recent publications have used single‐cell RNA‐seq to identify previously unrecognised macrophage populations with unique gene expression signatures.^87^, ^88^, ^89^, ^90^, ^91^ These new subtypes may represent macrophage adaptation to unique microenvironments within organs. Macrophage subtypes are also classified based on the expression of few cell surface markers, but M1 or M2 macrophages can acquire canonical markers of the other subset *in vitro*.^92^, ^93^ In spite of the M1/ M2 classification model being accepted as an over‐simplification, it continues to be widely used.^94^ A standardised experimental framework for macrophage subtype classification based on the source of macrophages, activators used and macrophage markers has been proposed.^95^ Single‐cell RNA sequencing, mass cytometry and advanced clustering algorithms should shed more light on macrophage heterogeneity in the future.^96^, ^97^

## The macrophage niche

Tissue‐resident macrophages develop with the organ they reside in and adapt to perform not only immune functions but also homeostatic functions essential for the particular organ they inhabit.^12^, ^98^ Circulating monocytes taking up residence in tissues also adopt a tissue‐specific identity very similar to resident macrophages, if not completely similar.^99^ The existence of a niche for macrophages in individual tissues has been postulated. These niches may nurture and modify macrophages by providing them with a physical scaffold and trophic factors for survival and proliferation. The type of physical scaffold may affect the differentiation and function of macrophages by inducing specific transcription factors to suit the temporal homeostatic function of a tissue.

## Niche adaptation of macrophages

**Figure 2:**
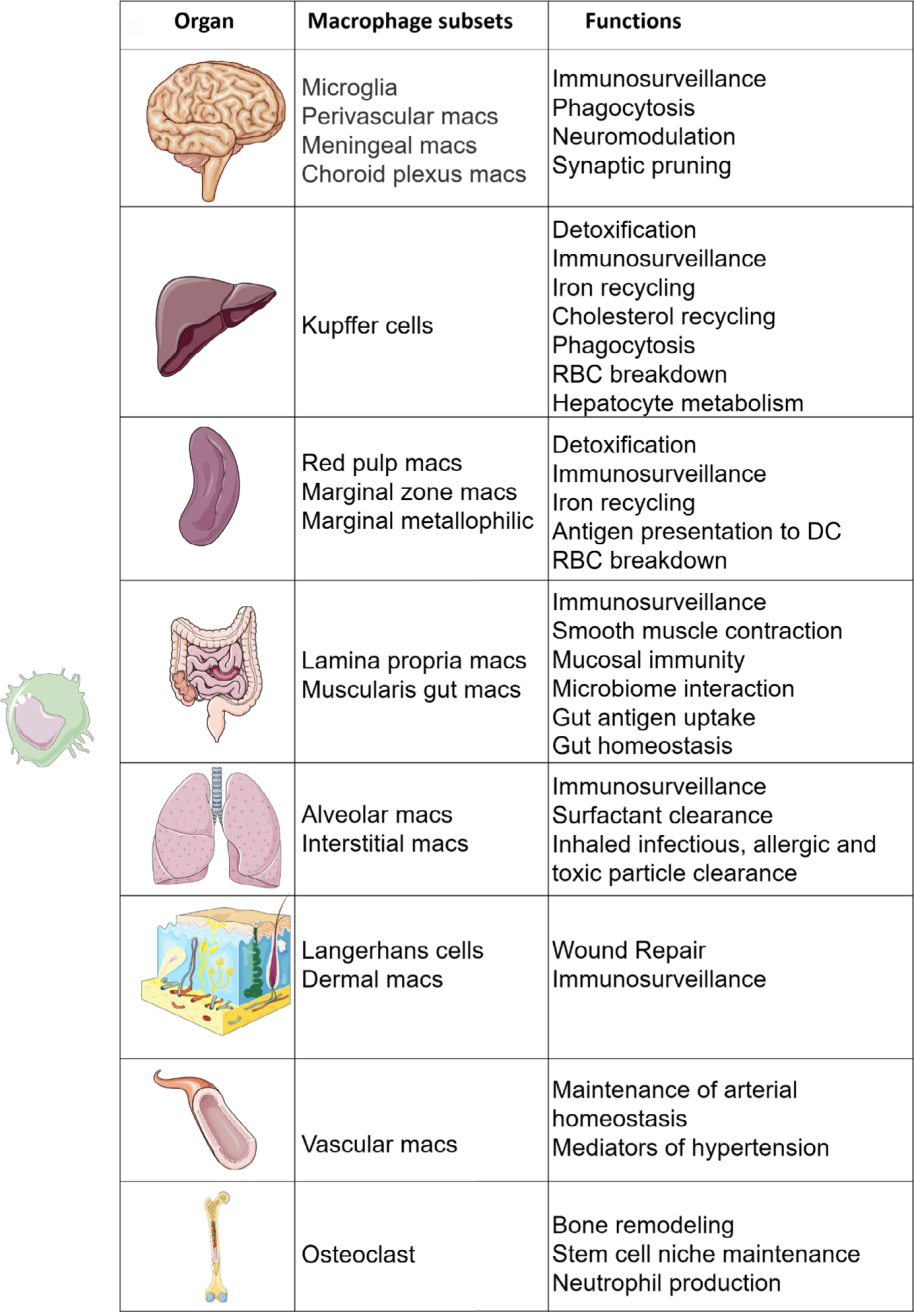
The heterogenous functions of tissue macrophages. All tissue macrophages go through a process of lineage determination via expression of limited set of transcription factors to acquire functions and cell surface markers common to all macrophages (phagocytosis, F4/80, MertK). The tissue microenvironment customises the macrophage to take over organ‐specific functions by inducing expression of unique set of transcription factors. Multiple signals specific to a tissue in a sequential combination are required to prime the macrophage and prepare the epigenetic landscape for macrophages to take up a tissue‐specific identity.

All tissue macrophages, after going through a programme of lineage determination directed by a unique set of transcription factors such as PU.1 and MafB,^54^, ^100^, ^101^, ^102^ acquire a common set of functions (e.g. phagocytosis, immune surveillance) and cell surface markers (F4/80, CD64, Mertk). Ultimately, the tissue microenvironment customises the local macrophage population to suit its homeostatic needs (Figure 2). As organogenesis proceeds, the differentiating *milieu* of an organ guides the resident macrophages to acquire the phenotype and functions appropriate to that organ. Expression of a limited set of transcription factors confers a tissue‐specific character on macrophages. For example, nuclear factor of activated T cells 1 (NFATC1) is necessary for osteoclast differentiation and functional specialisation.^103^ Similarly, transforming growth factor‐ β (TGF‐β) signalling,^104^, ^105^, ^106^, ^107^, ^108^ notch signalling^109^, ^110^, ^111^, ^112^ and bone morphogenetic protein (BMP) signalling drive the specialisation of multiple subsets. It appears that all macrophage subsets are active phagocytes and the material they ingest appears to dictate their fate. Tissue macrophages are exposed to specific metabolites in different organs. For example, haem,^113^ oxysterol^113^, ^114^, ^115^ and retinoic acid^98^, ^116^, ^117^ can induce functional polarisation of macrophages. Macrophage crosstalk with other immune cells also plays a role in defining their differentiation. For example, alveolar macrophage development involves crosstalk with pulmonary innate lymphoid cell 2s (ILC2s) and basophils producing CSF2 and IL‐13,^118^ whereas LC replenishment requires CSF1 produced by neutrophils.^119^

Even though a restricted set of transcription factors decides macrophage identity, multiple signals in a specific sequence are required to prime macrophages and prepare the epigenetic landscape for macrophages to adopt a tissue‐specific identity. Predictably, these multiple signals are specific to a location or a macrophage niche. For example, monocyte engraftment to liver requires interaction with endothelial cells, hepatocytes and stellate cells with key roles for TGF‐β and desmosterol.^109^, ^112^ Another example of multistep imprinting of macrophage identity by the niche is mucosal Langerhans cell differentiation. In this particular example, precursor cells have to be first exposed to BMP7 in lamina propria and then TGF‐β from endothelial cells to complete their differentiation.^120^, ^121^ Thus, a unique combination of tissue niche factors can induce reversible activation of gene expression programmes that is responsible for functional polarisation of macrophages in tissues.

## Nurture in the niche

Macrophages require a continuous supply of trophic factors IL‐34, CSF‐1(M‐CSF) and CSF‐2 (GM‐CSF) for normal maintenance and development. IL‐34 and CSF‐1 share a similar tertiary structure and bind to a common receptor, CSF1R.^5^, ^122^, ^123^, ^124^, ^125^ CSF‐1 is produced in three different forms by alternate splicing (secreted form, secreted proteoglycan form and a membrane form), and all three forms have a common active N‐terminal and distinct but overlapping functions.^126^, ^127^, ^128^ CSF‐1‐deficient mice have normal number of Langerhans and microglial cells but display a deficiency in most other macrophage types.^53^ Reconstitution of CSF‐1‐deficient mice with the soluble form of CSF‐1 rescues most resident macrophage populations, the membrane form corrects for macrophages in most tissues except in liver, spleen and kidneys.^127^, ^128^ The proteoglycan form of CSF‐1 integrates into the matrix of local tissues and regulates local macrophage numbers. Mice lacking CSF1R, the common receptor for IL‐34 and CSF‐1, have reduced numbers of macrophages throughout the body.^53^, ^129^ Administration of anti‐CSFR1 blocking antibody depletes most of the macrophage populations in embryonic^67^ and adult tissues.^130^ conversely, administration of CSF‐1 produces a massive expansion of blood monocytes and tissue macrophages in mice.^131^, ^132^, ^133^, ^134^ The secreted form of CSF‐1 when injected into CSF‐1‐deficient mice rescues most of the resident macrophage population.^127^, ^128^ In fact, administration of CSF‐1 leads to increased macrophage numbers in the liver and a rapid increase in the size of liver.^131^, ^134^ This may indicate a role for macrophages in homeostatic regulation of organ size. CSF‐1 consumption by Ly6C^hi^ monocytes can regulate the generation of the Ly6C^lo^ subpopulation^17^, ^125^, ^132^ and depletion of monocytes can lead to an increase in circulating CSF‐1 levels which, in turn, will promote an increase in tissue macrophage numbers. Bioavailability of CSF‐1 may also be regulated by post‐translational modification. For example, tumor necrosis factor‐α (TNF‐α)‐converting enzyme (TACE) can convert the membrane‐bound isoform of CSF‐1 to the soluble form of CSF‐1.^135^ IL34 KO mice are deficient in LC and brain microglia.^136^, ^137^, ^138^, ^139^, ^140^ IL‐34 is not detected in blood, probably because it acts locally near the tissue where it is produced.^141^, ^142^ In summary, IL‐34 and CSF‐1 are the most important trophic factors produced by the macrophage niche and are essential for the maintenance and survival of macrophages.

## Regulation of macrophage density in tissues

Resident macrophages are abundant in every organ of the body and have similar relative densities and are arranged with regular spacing.^143^, ^144^ The regular spacing of macrophages in tissues has been explained by self‐avoidance or self‐repulsion.^145^ Macrophages may actively surveil large areas^146^ of their environment through highly motile filopodia^145^ and actively repel neighbouring macrophages when encountered. Thus, macrophages may establish territories in a cell‐autonomous manner. The mutual repulsion theory may not be an entirely sufficient explanation, as macrophages are densely packed in splenic red pulp and the subcapsular sinus of the lymph node,^113^, ^147^, ^148^ compared to the T‐cell zone of these two organs where macrophages are regularly patterned.^149^ Thus, the repulsion hypothesis may not be sufficient to explain macrophage density in some organs, with other variables such as tissue‐specific factors or inflammatory status playing a role in macrophage density. Zhou *et al*.^150^ used the concept of carrying capacity from evolutionary biology to postulate that each tissue has an abundant population of cells like fibroblasts whose numbers are regulated by the carrying capacity of that tissue (the carrying capacity of a tissue is influenced by the availability of glucose, oxygen, space and other growth factors). The abundant tissue fibroblast population can then in turn negatively regulate an accessory population of cells, such as macrophages. Fibroblasts form a cell‐circuit based on growth factor exchange with macrophages.^150^, ^151^ Fibroblasts produce the macrophage survival factor CSF‐1, whilst macrophages provide the fibroblast growth factor PDGFs. Both CSF‐1 and its receptor (CSF1R) are rapidly internalised upon binding allowing for negative feedback regulation of macrophage numbers. This reductionist explanation may also provide a template for complex models involving multiple cell types, secreted factors and physical interactions coming together to regulate macrophage density in tissues. Also, since macrophage numbers are well below the carrying capacity of tissues, inflammation may transiently change the status quo and lead to increases in the number of macrophages.

During inflammation, apoptotic macrophages may produce chemotactic factors to attract monocytes which would then clear the dying cell and occupy the vacant site.^49^, ^152^, ^153^ Another possibility is that an increase in local concentration of tropic factors (e.g. CSF‐1) after macrophage death may cause neighbouring macrophages to divide and occupy the available space.^154^ Yet, another possibility is that inflammatory conditions may lead to downregulation of macrophage‐repulsive function and increase their number in tissues. The degree of inflammation is correlated with the engraftment efficiency of infiltrating monocytes.^109^ During Listeria infection, the recruitment and differentiation of monocytes into KCs are regulated by the release of IL‐1 from dying KCs.^155^ The time window of inflammation may also affect infiltrating monocyte engraftment vs. repopulation by dividing tissue macrophages, as the infiltrating monocytes are at a disadvantage because they must differentiate into macrophages before they can engraft. This may be the reason why tissues in a state of constant inflammation witness the highest turnover rate of tissue macrophages. For example, infiltrating monocytes replace gut macrophages only after the establishment of gut microbiota^156^ and the contribution of infiltrating monocytes to gut macrophages is very low in antibiotic‐treated and germ‐free mice.^156^, ^157^ Monocyte‐derived macrophages also gradually replace tissue macrophages in organs (kidney, heart, liver) that are subject to continuous low‐grade inflammation because of mechanical or metabolic inflammation^158^, ^159^ and levels of monocyte‐derived macrophages in tissues may be an indicator of the inflammatory state of specific tissue.^160^ Thus, in summary each tissue has a certain macrophage density under homeostatic conditions that can be substantially altered by inflammatory conditions.

## We are what we eat: immunometabolism of macrophages

Metabolic pathways contribute to the development, fate and behaviour of macrophages and are critical for induction of inflammatory responses and initiation of tissue healing.^13^, ^161^, ^162^, ^163^ The plastic nature of macrophages is reflected in the ability of macrophages to make dramatic changes to their intracellular metabolism in response to environmental and inflammatory cues. In macrophages treated with lipopolysaccharide (LPS), prototypical of inflamed macrophages, the Warburg effect is observed with a preference towards glycolysis over oxidative phosphorylation.^164^ Inflammatory activation of macrophages by LPS hampers pyruvate transport to the mitochondria and inhibits the TCA cycle. Pyruvate generated during glycolysis is preferentially converted to lactate instead of being shuttled into mitochondria to be converted into acetyl‐CoA to fuel the tricarboxylic acid cycle (TCA) cycle. The many inflammatory stimuli [e.g. pathogen‐associated molecular patterns (PAMPs) and damage‐associated molecular patterns (DAMPs)] that activate NF‐κB also lead to activation of HIF1α which in turn causes macrophages to switch to glycolysis and inhibit TCA cycle.^165^

The preference for glycolysis is conducive for an inflammatory response in macrophages. Glycolysis is not only a faster source of ATPs but also has other roles during inflammation. For example, lactate produced by glycolysis is involved in termination of inflammation.^166^ Increased lactate promotes histone acetylation that leads to arginase 1 expression and resolution of inflammation.^166^, ^167^ The pentose phosphate pathway which is highly activated in a glycolytic cell provides ribose sugars and NADPH for biosynthetic pathways essential for macrophage inflammatory response.^75^, ^168^ LPS inhibits the expression of SHPK (sedoheptulose kinase) that controls the non‐oxidative phase of the pentose phosphate pathway. This inhibition increases the availability of ribose to be used for fatty acid and sterol synthesis pathways. The enhanced commitment to glycolysis in activated macrophages also supports the production of inflammatory mediators (e.g. TNF‐α, CCL2, IL‐12 and nitric oxide), and these mediators in turn have an inhibitory effect on critical steps of the TCA cycle.^168^ In macrophages, LPS boosts the expression of several rate‐limiting enzymes in glycolysis, including hexokinase 3,^169^ PFKFB3 (6‐phosphofructo‐2‐kinase/ fructose‐2,6‐biphosphatase 3) and pyruvate kinase isozymes 2 (PKM2).^169^, ^170^ These changes are coupled to the inhibition of the key TCA cycle enzymes, isocitrate dehydrogenase and succinate dehydrogenase leading to accumulation of citrate and succinate.^168^, ^171^, ^172^ The autocrine type I IFN pathway is responsible for the inhibition of isocitrate dehydrogenase in LPS‐stimulated macrophages.^173^ Nitric oxide (NO) produced by M1 macrophages can also lead to suppression and loss of mitochondrial electron transport chain (ETC) complexes and rerouting of pyruvate away from pyruvate dehydrogenase (PDH) to promoting glutamine‐based anaplerosis.^174^ These TCA cycle intermediates get diverted to other biosynthetic reactions specific to inflammatory metabolism. The full spectrum of inflammatory activation by macrophages requires increased expression of glycolytic enzymes and accumulation of TCA cycle intermediates. For example, hexokinase is needed for inflammasome activation and release of IL‐1β^175^, ^176^; similarly, PKM2 serves to increases glycolytic flux by induction of GLUT‐1 in the nucleus and serves as a co‐activator for HIF1α.^177^ PKM2/HIF1α complexes bind to the *Il1β* promoter and induce IL‐1β expression.^178^ The accumulation of certain metabolites can support macrophage activation or restore homeostasis. Succinate, can drive IL‐1β production *via* stabilisation of HIF1α,^172^ whereas citrate accumulation can, via malonylation of GAPDH, promote TNF‐α translation.^179^

In addition to LPS, the effect of other factors on the metabolism of macrophages and their inflammatory status has been studied. For example, insulin has been shown to enhance glycolysis and IL‐1β secretion in intraperitoneal macrophages.^180^ IL‐1β is also known to activate macrophages in the pancreas leading to β‐cell dysfunction and death,^181^ explaining a link between chronic elevation of IL‐1β signalling and type 2 diabetes.^182^ Indeed, in patients with type 2 diabetes, blockade of interleukin‐1 with IL‐1 receptor antagonist anakinra improved glycemia and β‐cell secretory function and reduced markers of systemic inflammation.^183^ Macrophages exposed to oxidised phospholipids in hyperlipidemic states use mitochondrial respiration, feeding the Krebs cycle with glutamine and causing the accumulation of oxaloacetate in the cytoplasm. This subsequently leads to increased IL‐1β production, resulting in hyperinflammation.^184^ Oxidised LDL also can bind to CD36 on macrophages and suppress oxidative phosphorylation leading to mitochondrial ROS production, which drives chronic inflammation.^185^ Macrophages exposed to extracellular pathogenic lipids can activate a triggering receptor expressed on myeloid cells 2 (TREM2)‐dependent gene response involved in phagocytosis and lipid catabolism.^186^, ^187^ TREM2 expression is required for a metabolic switch towards glycolysis and is essential for the maintenance of healthy energy metabolism under conditions of stress.^90^, ^188^ TREM2 signalling also drives the formation of lipid‐associated macrophages (LAM) in adipose tissue. LAMs regulate systemic lipid homeostasis in obesity^90^ and may also be involved in suppression of obesity‐induced inflammation.^90^ TREM2 macrophages are also reported to play a role in neurodegenerative disease^189^ and atherosclerosis.^87^ Hypoxia can induce glycolysis in macrophages, for example tumor‐associated macrophages (TAMs) present in the hypoxic regions of tumors express HIF‐1α inducing a switch to glycolytic fermentation. High amounts of lactic acid present in the tumor microenvironment also stabilise the expression of HIF‐1α and cause M1 to M2 polarisation.^167^ Hypoxia also promotes pro‐tumoral activities of TAMs by increasing the availability of iron for tumor cell proliferation and by causing upregulation of DNA damage‐inducible transcript 4 (DDT4), which inhibits the mechanistic target of rapamycin (mTOR) pathway to promote OXPHOS and reduced glucose intake in TAMs.^190^

In contrast to LPS‐treated macrophages, IL‐4‐treated M2 macrophages are more dependent on OXPHOS and have an intact TCA cycle.^191^ The elevated OXPHOS in M2 macrophages is supported by increased FAO.^77^ There is some debate as to the role of glycolysis in M2 macrophages.^192^ However, both glucose and glutamine seem to support OXPHOS and M2 polarisation.^193^ Macrophage activation by IL‐4 stimulates the Akt‐mTORC1 pathway which regulates ATP citrate lyase (ACLY), a transferase that catalyses the conversion of citrate and coenzyme A to acetyl‐CoA, leading to increased histone acetylation and M2 gene induction.^194^ In comparison, the impaired OXPHOS in LPS‐treated macrophages can reduce acetyl‐CoA levels and alter histone acetylation, leading to impaired expression of inflammatory genes and tolerance.^195^ LPS stimulation of macrophages also results in reduction of FAO,^168^ whereas IL‐4 can induce FAO through transcription factors STAT‐6 and PGC1β.^76^ In summary, macrophage plasticity is most likely supported by their remarkable ability to remodel their core metabolic pathways in response to a range of signals. This rewiring of metabolism provides a faster source of energy, activates biosynthetic pathways needed for inflammation, stimulates the production of inflammatory mediators such as IL‐1β and TNF‐α and sets the ground for shutdown of inflammation in a time‐delayed manner.

## The relationship between monocytes and macrophages

No discussion of macrophages is complete without an understanding of the origin and function of monocytes. Monocytes can give rise to macrophages under pathological conditions and can support near‐complete reconstitution of tissue macrophages after depletion. Human monocytes are divided into three groups based on the expression of CD14 and CD16 on HLA‐DR^+^ cells. CD14^+^CD16^−^ monocytes are referred to as classical monocytes, CD14^+^CD16^+^ cells as intermediate cells and CD14^‐^CD16^+^ monocytes are referred as non‐classical monocytes. Mouse monocytes are divided into Ly6C^hi^ monocytes (also defined as CX3CR1^int^ CCR2^+^ CD62L^+^ CD43^lo^) and Ly6C^lo^ monocytes (CX3CR1^hi^ CCR2^−^ CD62L^−^ CD43^hi^).^34^, ^72^, ^81^, ^196^ Transcriptional comparisons correlate mouse Ly6C^hi^ monocytes with ‘classical’ CD14^+^CD16^−^ monocytes in humans and Ly6C^lo^ monocytes with ‘non‐classical’ CD14^lo^ CD16^+ ^monocytes. Despite similar transcriptional profiles and cell surface marker expression, differences exist between human and mouse monocytes. For example, major histocompatibility complex (MHC II) is expressed on Ly6C^lo^ monocytes and absent on mouse Ly6C^hi^ monocytes, but human monocytes overall are positive for MHC II.^197^

## Monocyte origins and egress from BM

According to the classical model of monocyte development, monocytes arise from haematopoietic stem cell‐derived common myeloid progenitor (CMP) with granulocyte–macrophage progenitors (GMPs), macrophage (monocyte)/ dendritic cell precursor (MDP) and common monocyte progenitor (cMoP) acting as intermediates.^198^ Yanez et al. showed that MDPs arise directly from CMPs directly and give rise to monocytes via cMoPs.^199^ Also recently, Liu *et al*.^200^ used Ms4a3 reporter mice (a specific gene reporter for GMPs) and showed that MDPs do not arise from GMPs and that monocytes arise from both GMP and MDPs. Emergency monopoiesis can also give rise to granulocyte like segregated nucleus containing Ly6C^lo^ monocytes (SatM).^201^ Ly6C^hi^ monocytes egress out of BM in a CCR2/CCL2/CCL7.^202^, ^203^, ^204^ and CXCR4 ‐dependent manner.^205^, ^206^, ^207^ CCL2 and CCR2‐deficient mice show increased number of monocytes in the BM but fewer numbers in the periphery.^202^ Ly6C^hi^ monocytes in the BM parenchyma are juxtaposed to nestin^+^ stromal cells.^206^, ^208^ CCL2 binding to CCR2 leads to desensitisation of monocyte response to CXCL12 because of internalisation of CCR2‐CXCR4 complex, which weakens CXCR4 binding and causes egress of monocytes out of BM.^206^, ^209^ The release of Ly6C^hi^ monocytes from BM is also regulated by circadian rhythm. Ly6C^hi^ monocyte egress from BM peaks between 4 and 8 hours after light onset and is controlled by the circadian rhythm transcription factor, Bmal1.^210^ The number of circulating monocytes is strongly linked to the physiological status of an organism^211^ and depends on monocyte production and release from BM and peripheral reservoirs. Exercise, age and a host of other pathophysiological conditions (e.g. chronic inflammatory disorders) can also influence the number and ratio of monocyte subsets.^212^, ^213^, ^214^, ^215^

## Monocyte reprogramming or conversion to macrophages

Classical monocytes (Ly6C^hi^ monocytes in mice) have a diverse differentiation potential because of their plastic transcriptional profile which allows them to take on different roles under homeostatic conditions. Classical monocytes comprise over 90% of circulating monocytes,^81^ and upon extravasation into tissues, they contribute to the innate immune response via production of TNF‐α and NO, or by differentiating into macrophages and dendritic cells.^72^ Ly6C^hi^ monocytes can replace embryo‐derived tissue‐resident macrophages by differentiating into macrophages.^69^, ^222^ Conversion of monocyte to tissue macrophages is accompanied by extensive transcriptional changes to mirror the transcriptome of resident macrophages. Even though monocyte‐derived macrophages adopt most of the functions associated with the tissue‐resident macrophages that they are replacing, some epigenetic, transcriptional and functional differences remain.^11^, ^99^, ^223^, ^224^ Some monocytes can also remain within tissues, show minimal transcriptional change and act as a local monocyte reservoir.^225^ These monocytes can survey resident tissues and transport antigen to lymph nodes.^226^ Once in the lymph nodes, they can either differentiate into dendritic cells or remain as monocytes while losing their ability to recirculate.^72^ Thus, monocytes as macrophage precursor cells that mirror the flexibility and plastic nature of macrophages can readily replace tissue macrophages.

## Tissue‐Resident Macrophages and the Relevance of MPS Classification

Monocytes are rapidly recruited to sites of inflammation/injury and depending on the situation they encounter they undergo different cell fates. Under conditions of inflammation, tissue injury or macrophage depletion, embryonically derived macrophages undergo death and are replaced by monocyte‐derived macrophages.^69^, ^71^, ^155^, ^227^, ^228^ Long‐term integration of monocyte‐derived macrophages depends on the type of tissue and conditions encountered. For example, monocyte‐derived macrophages do not integrate into the CNS after injury,^64^ but do integrate into the heart with ageing and after a myocardial infarction (MI).^220^, ^227^ They also integrate into the peritoneal cavity after thioglycolate challenge^17^ and in the liver after KC depletion.^224^ Under inflammatory conditions, they take on pro‐inflammatory effector functions and DC‐like functions such as antigen presentation and migration to LNs. In addition to monocyte‐derived macrophages, peritoneal cavity macrophages and pericardial macrophages can also be recruited to sites of inflammation. For example, Gata6^+ ^peritoneal cavity macrophages are recruited to help resolve inflammation in the setting of sterile liver injury,^229^ and Gata6^+^ macrophages in mouse pericardial fluid contribute to reparative immune response in heart following experimental MI.^230^ Since these macrophages do not have to take a vascular route to get to the sites of injury or undergo differentiation into macrophages, they may represent rapid responders to the site of injury.

Tissue‐resident macrophages are imprinted to have a higher tolerance to stimuli associated with acute inflammation, while macrophages derived from infiltrating monocytes may be more inflammatory. In experimental autoimmune encephalomyelitis (EAE) which is a commonly used murine model for multiple sclerosis, infiltrating monocytes trigger EAE progression.^18^ Monocyte‐derived macrophages in EAE are highly phagocytic, express pro‐inflammatory genes such as IL‐1β and TNF‐α^231^, ^232^ and initiate demyelination, whereas microglia are inert and appear to be dedicated to the clearance of debris.^231^ CCR2‐deficient animals (deficient for recruitment of Ly6C^hi^ monocytes) and mice depleted for Ly6C^hi^ monocytes are relatively protected from EAE.^233^ Monocyte‐derived cells and microglia remain distinct entities during disease progression. Following recovery, recruited monocytes vanish and do not integrate into the resident microglial pool, while the microglia can enter the cell cycle and return to quiescence following remission from EAE.^64^ Such a scenario, where tissue‐resident macrophages have higher inflammatory signal threshold, is also supported by studies focusing on acutely inflamed gut^218^, ^234^ and liver.^235^ Monocyte‐derived macrophages also replace the Kupffer cells lost because of inflammation in nonalcoholic steatohepatitis (NASH) a form of nonalcoholic fatty liver disease. A NASH diet was found to induce significant changes in resident Kupffer cell gene expression and result in cell death, while monocyte‐derived macrophages replacing the lost Kupffer cells exhibited convergent epigenomes, transcriptomes and functions.^187^

Gut macrophages are the largest macrophage population in the mouse, and macrophages in the intestinal lamina propria are continuously replaced by blood monocytes in the adult mouse.^157^ TGF‐β‐dependent monocyte differentiation in the colonic lamina propria causes rapid downregulation of inflammatory signalling molecules and rapid upregulation of receptors involved in apoptotic cell recognition.^107^ However, the intestine has a population of TIM4^+^CD4^+^ macrophages that can self‐maintain for months.^157^

The liver and lung macrophage populations are seeded primarily from foetal liver‐derived monocytes and maintained by self‐renewal.^43^, ^67^ Depletion of KCs in adult mice would result in BM‐derived monocytes occupying the vacant sinusoidal location and adopting the transcriptomic profile and clearance functions of the cells they replaced.^224^, ^236^, ^237^ YS macrophages, foetal liver monocytes or adult BM monocytes when transplanted to *Csf2r* ^−/−^mice can acquire the differentiated alveolar macrophage phenotype.^99^ When all the three subtypes were mixed and transferred to *Csf2r* ^−/−^ mice, preferential outgrowth of foetal monocytes was observed, correlating with better GM‐CSF sensitivity. When transferred separately, however, all precursors colonised the alveolar niche and generated AMs that were transcriptionally almost identical and self‐maintained.^99^

Brain macrophage populations are established during embryonic development and are maintained independently of monocytes. Microglia are constantly replaced by proliferation in the adult mouse brain,^152^ and the perivascular macrophages have also been shown to be maintained independently of monocytes.^153^ However, donor monocytes could replace brain macrophages in irradiated mouse chimeras,^49^, ^153^ macrophage‐deficient, PU.1 knockout mice^238^ and *Csf1r* ^–/–^ mice at birth.^239^ As in liver, subtle transcriptome differences are detected between resident microglia and the engrafted macrophages.^223^

Cardiac macrophages originate from YS and foetal monocyte progenitors, and four different types of macrophages have been reported.^69^, ^220^ Monocyte‐derived macrophages increase in the heart with age, and Ly6C^hi^ monocytes were able to differentiate into long‐lasting populations of cardiac macrophages after macrophage depletion.^220^ F4/80^hi^ peritoneal macrophages are also slowly replaced by differentiation of F4/80^lo^ MHCII^+^ monocyte‐derived progenitors. All the above evidence points to the continued relevance of the MPS model where blood monocytes can and do enter tissues to progressively replace tissue macrophages.

## Conclusion

Macrophages are key players in the immune system, but beyond their role as sentinels, macrophages play a crucial role during development and homeostasis. After starting out with a relatively homogenous gene expression profile in the embryo, macrophages become specialised for disparate functions in different tissues. This diversity of function makes us rethink their classification as a single‐cell type. Despite functional specialisation in different locations, macrophages are amazingly plastic with a fluid identity. Monocyte‐derived macrophages further add to the complexity by functionally replacing embryo‐derived macrophages but still retaining a lower threshold for inflammatory activation and not quite taking over the reparative function of tissue‐resident macrophages. Much still needs to be understood regarding the origin and maintenance of tissue‐resident macrophages. We need to fully understand the reparative properties of embryo‐resident macrophages and why they are progressively lost with age and why monocyte‐derived macrophages are lacking in their reparative capability. Exploiting these diverse macrophage functions for therapeutic benefit is a promising strategy in a range of pathologies.

## Funding

This work was supported by NIH funding to PRN (HL137799).

## Conflicts of interest

The authors declare no conflicts of interest.

## Author Contribution

**Andrew Fleetwood:** Writing‐review & editing. **Andrew Murphy:** Conceptualization; Writing‐review & editing. **G Sreejit:** Conceptualization; Writing‐review & editing. **P Nagareddy:** Conceptualization; Writing‐review & editing.
